# Recombinant human erythropoietin promotes the acquisition of a malignant phenotype in head and neck squamous cell carcinoma cell lines in vitro

**DOI:** 10.1186/1756-0500-4-553

**Published:** 2011-12-21

**Authors:** Eric Abhold, Elham Rahimy, Jessica Wang-Rodriguez, Katherine J Blair, Michael A Yu, Kevin T Brumund, Robert A Weisman, Weg M Ongkeko

**Affiliations:** 1 Department of Pathology, University of California, San Diego and the VA San Diego Healthcare System, San Diego, CA, USA; 2 Division of Otolaryngology-Head and Neck Surgery, Department of Surgery, University of California, San Diego, San Diego, CA, USA; 3 University of California, San Diego, Biomedical Sciences Building Rm. 1202 Mail Code 0612 9500 Gilman Dr, La Jolla, CA92093-0612, USA

## Abstract

**Background:**

Recent studies indicate an increase in tumor progression and recurrence in head and neck squamous cell carcinomas (HNSCC) of cancer patients taking recombinant human erythropoietin (rhEpo) for anemia. This study was undertaken to investigate the potential role of rhEpo in invasion, proliferation, and cisplatin-induced cell death in HNSCC cell lines.

**Methods:**

The following experiments were performed with two HNSCC cell lines, UMSCC-10B and UMSCC-22B. Presence of EpoR in both cell lines was determined by western blot and quantitative PCR. Colorimetric MTS assays and clonogenic assays were used to study the effect of rhEpo at pharmacologically relevant doses on cell proliferation. Matrigel invasion assays were performed in order to determine effects of exogenous rhEpo on invasive abilities. Clonogenic assays were also used to study potential cytoprotective effects of rhEpo against cisplatin. Immunoblotting was done to analyze the effect of rhEpo on Akt phosphorylation. Finally, MTS and TUNEL assays were performed to test our hypothesis that Akt activation by PI3K was involved in rhEpo-mediated cisplatin resistance.

**Results:**

HNSCC cell lines were shown to express Epo receptor (EpoR). RhEpo increased invasion 1.8-fold in UMSCC-10B and 2.6-fold in UMSCC-22B compared to control. RhEpo at 10 U/ml increased cell proliferation by 41% and 53% in UMSCC-10B and UMSCC-22B, respectively, and colony formation by 1.5-fold and 1.8-fold. UMSCC-10B treated with cisplatin and exposed to rhEpo at 1 and 10 U/ml resulted in a 1.7-fold and 3.0-fold increase in colony number compared to control, respectively. UMSCC-22B treated with cisplatin and rhEpo at 1 or 10 U/ml resulted in ~2.5-fold increase in colony number. A TUNEL assay demonstrated a 30.5% and 76.5% increase in survival in UMSCC-10B and UMSCC-22B cells, respectively, in cisplatin and rhEpo-treated cells compared to cisplatin alone. MTS assay showed similar cytoprotective effects. Western blot revealed increased phosphorylation of Akt upon exposure of HNSCC cell lines to rhEpo. MTS assay and TUNEL analyses implicate Akt as a likely contributor to regulation of rhEpo-mediated cytoprotection.

**Conclusions:**

The results demonstrate that, in HNSCC cells expressing functional EpoR, rhEpo promotes invasion, cell proliferation, and induces resistance to cisplatin, which may contribute to tumor progression.

## Background

Erythropoiesis stimulating agents (i.e., recombinant human epoetin alfa) have been widely used to treat anemia. Recombinant human epoetin alfa (rhEpo) is a glycoprotein (30.4 kDa) produced by recombinant DNA technology, and has the same biologic effects as the endogeneous erythropoietin produced by the kidneys. RhEpo has been used since 1993 for the treatment of anemia, including those associated with chemo- and radiation therapy in cancer patients. Early on, it was thought that rhEpo exerts its effect(s) exclusively in hematopoietic tissues, where it plays a crucial role in the maturation of red blood cells. However, recent studies have shown expression and function of Epo and EpoR in a variety of human cancers, including solid tumors and tumor cell lines [1-3]. As such, treatment with rhEpo could have unintended pharmacologic consequences. Given the precise role of rhEpo in human cancers, particularly tumor progression and recurrence, is not well understood, clinical and basic research studies are still necessary to define signaling pathways activated by rhEpo/EpoR within nonhematopoietic cancer cells.

The presence of EpoR in cancer tissues, if functional, could have unintended consequences in patients who use rhEpo for radiation- and chemotherapy-associated anemia. In 2003, major safety issues with ESA administration in breast cancer patients undergoing chemotherapy were reported when a clinical trial was terminated early because of increased mortality risks [4]. Similar safety issues were subsequently reported in another clinical trial involving patients with head and neck squamous cell carcinoma (HNSCC) undergoing radiotherapy [5]. In both trials, poor survival was identified for patients who were treated with ESAs, mainly due to early disease progression [4,5]. Six additional trials observed adverse outcomes, such as decreased survival and locoregional disease control, in ESA-treated patients with a wide range of malignancies including lymphoid, cervical, non-myeloid, and non-small cell lung cancer [6]. In four of the eight aforementioned studies, patients received chemotherapy or radiation therapy [6]. These findings emphasize the need to understand the role of rhEpo/EpoR signaling in cancers and evaluate the use of rhEpo in cancer patients carefully.

More recently, a meta-analysis, utilizing data from clinical trials evaluating erythropoiesis stimulating agents (ESAs, as a product class) for the treatment of anemia in the oncology setting, has further analyzed the risks of mortality associated with administration of ESAs for anemia in cancer patients [7,8]. The results of the analysis indicated increased mortality when ESAs were administered to cancer patients with anemia. This finding is consistent with those reported in clinical trials that have prospectively evaluated survival, as a primary or secondary outcome measure, and individually identified increased rates of mortality or tumor progression with the use of ESAs [7].

These major safety issues have prompted the FDA to restrict the use of ESAs for the treatment of anemia in cancer patients, adding 'Warnings' to ESAs approved labelling information. These safety issues have also necessitated further studies into the underlying mechanisms by which ESAs lead to poorer survival of cancer patients.

There are published reports indicating that exogenously administered and endogenously expressed Epo can induce cellular invasion, promote cell proliferation and inhibit apoptosis [9-11], but the precise role by which rhEpo causes tumor progression in cancer patients is unclear. Therefore, further studies are necessary to evaluate the role of rhEpo/EpoR in human cancers. More specifically, rhEpo/EpoR potential functions have not been fully explored in HNSCC cells. We have undertaken studies to investigate whether (i) EpoR is expressed in established HNSCC cell lines; (ii) rhEpo promotes cell proliferation and invasion; (iii) rhEpo protects HNSCC cells from cisplatin-induced death, the first-line of chemotherapy treatment for this malignancy; and (iv) the PI3K/Akt signaling pathway is implicated in rhEpo-mediated HNSCC cisplatin resistance.

## Methods

### Drugs and reagents

Recombinant human epoetin-alfa (10,000 U/ml vial) was purchased from Amgen (Thousand Oaks, CA USA). Cisplatin (CDDP) was purchased from Sigma-Aldrich (St Louis, MO, USA) and a 3.33 mM stock solution was prepared in dimethyl sulfoxide (DMSO). PI3 kinase/Akt signaling inhibitor LY-294002 and Akt inhibitor IV were purchased from Sigma-Aldrich and freshly dissolved in DMSO at a stock concentration of 10 mM. Stock solutions were diluted in culture media to the indicated working drug concentrations prior to cell treatment. Control cells were treated with an equal volume of vehicle alone, and the concentration of DMSO in cell cultures never exceeded 0.5%.

### Cell lines and cell culture

Two established HNSCC cell lines, UMSCC-10B and UMSCC-22B, were gifts from Dr. Tom Carey, University of Michigan (Ann Arbor, MI, USA). Cell lines were cultured in DMEM supplemented with 10% fetal bovine serum (FBS), 2% streptomycin sulfate (Invitrogen, Carlsbad, CA), and 2% L-glutamine (Invitrogen), and maintained at 37°C in 5% CO_2 _and 21% O_2_.

### Real-time quantitative RT-PCR

At 90% confluence, cells were lysed and total RNA was extracted using an RNeasy Mini kit (Qiagen, Valencia, CA, USA). RNA was converted to cDNA using a Superscript III Reverse Transcriptase kit (Invitrogen, Carlsbad, CA, USA) as per the manufacturer's instructions. The levels of transcript for EpoR were quantified by real-time qPCR. The primers used were custom ordered (Operon Biotechnologies, Huntsville, AL, USA), and sequences were as follows:

EpoR Forward: 5'-CAAGTTCGAGAGCAAAGCGG-3',

EpoR Reverse: 5'-TTCCTCCCAGAAACACACCAAG-3';

β-actin Forward: 5'-ACAGAGCCTCGCCTTTGC-3',

β-actin-Reverse: 5'-CCTGGTGCCTGGGGC-3';

Reaction mixes were prepared as triplicates and run on the System 7300 Real-time PCR (Applied Biosystems) using a one-step program: 95°C for 10 min, 95°C for 30 s, and 60°C for 1 min, for 40 cycles. Results were analyzed by the relative quantity (ΔΔC_t_) method, and experiments were repeated at least twice independently. β-actin gene expression was measured as endogenous control.

### Western blot analysis

For baseline levels of EpoR, HNSCC cells were serum-starved for 24 h prior to protein extraction. To determine the effects of rhEpo on Akt phosphorylation, HNSCC cells were serum starved for 24 h prior to treatment with rhEpo at 1 U/ml for 3 or 72 h. At 90% confluence, cells were lysed in RIPA lysis buffer containing protease and phosphatase inhibitor cocktails. Total protein concentration was measured by a Bradford Protein Assay (Bio-Rad, Hercules, CA, USA) to enable standardization of protein loading. Lysate was separated on 10% SDS-PAGE gels, and electrophoretically transferred onto microporous polyvinylidene fluoride (PVDF) membranes (Millipore, Bedford, MA, USA) overnight at 40 V. Membranes were blocked with 5% BSA in tris-buffered saline with 0.1% Tween-20 (TBST), then incubated with the following primary antibodies, each at a 1:1,000 dilution, overnight at 4°C: rabbit anti-EpoR M-20 (Santa Cruz Biotechnology, Santa Cruz, CA, USA), mouse monoclonal anti-Epo 7D10 (Santa Cruz Biotechnology), mouse anti-β-actin (Sigma-Aldrich), rabbit total Erk (Cell Signaling, Beverly, MA, USA), and rabbit anti-phospho-Akt (Sigma-Aldrich). After a cycle of three 10-min washes with TBST, membranes were probed with the appropriate secondary antibody at 1:10,000 dilution at room temperature for 60 min. After 3 additional washes, the protein-antibody complexes were visualized by enzyme chemifluorescence (Pierce, Rockford, IL, USA).

### Matrigel invasion assay

Invasive properties of HNSCC cells were measured and compared in the presence or absence of rhEpo using Matrigel invasion assay (Becton Dickinson, Bedford, MA). Transwell inserts of 8 μm pore size were coated with 80 μl Matrigel (1 mg/ml) in cold serum-free DMEM. The lower chamber of the transwell was filled with 750 μl of culture media containing 0.5% serum as an adhesive substrate. Indicated treatments were also added to the lower chamber. Cells were trypsinized, and 500 μl of cell suspension (2 × 10^5 ^cells/ml) was added in triplicate wells and allowed to incubate at 37°C for 40 h. Invading cells on the lower surface that passed through the filter were fixed and stained using crystal violet in gluteraldehyde and photographed. The stained nuclei were counted (10 high-power fields per each chamber) and averaged for each treatment. Results are expressed as fold change in the number of invading cells for each treatment compared to control cells. Images were obtained using Leica DMIRE2 inverted fluorescence microscope. Computer program Simple PCI was used for image capture.

### Clonogenic survival assay

This assay was performed to assess potential effects of rhEpo on cell proliferation and against cisplatin-induced cell death in HNSCC. Cells were plated in triplicates at 500 cells per 60 × 15 mm culture plates and incubated in DMEM supplemented with 10% FBS, L-glutamine, and antibiotics. To test the hypothesis that rhEpo protects against cisplatin-induced cell death, UMSCC-10B and UMSCC-22B were serum starved for 24 h and treated with rhEpo at 0, 1 or 10 U/ml. Twenty-four hours later, the cells were exposed to 0.5 μM cisplatin for 72 h (UMSCC-10B) or 1.0 μM cisplatin for 96 h (UMSCC-22B). Cisplatin concentrations and incubation times were different for the cell lines, as these parameters were optimized for each. The media were replaced with complete media after the time periods indicated above, allowing the cells to recover and form colonies. Ninety-six hours later, the cells were fixed, stained, and colonies that contained over 50 cells were counted.

In addition, the effect of rhEpo on cell morphology after cisplatin treatment was determined by light microscopy. HNSCC cell lines were grown on cover slips, then pre-treated with rhEpo at 1 U/ml for 24 h prior to the addition of cisplatin for 48 h. Cells were fixed with methanol and images were obtained using Leica DMIRE2 inverted fluorescence microscope. Computer program Simple PCI was used for image capture.

### MTS assay

To assess effects of rhEpo on cell proliferation, logarithmically growing HNSCC cells were trypsinized, washed, and seeded in 96 well plates at low cell density (1,000 cells/well). After allowing the cells to adhere overnight, varying concentrations of rhEpo (1, 10, 20 U/ml) were added to the medium in serum free conditions for 6 days. To investigate the role of PI3K/Akt in rhEpo-mediated cisplatin resistance, cells were plated at high density (6,000 cells/well) and allowed to adhere overnight. Cells were maintained in serum free conditions then treated with or without the PI3K/Akt signaling inhibitor LY-294002 (10 μM) or Akt inhibitor IV (1.25 μM) for 60 min prior to treatment with rhEpo at 10 U/ml. After 24 h, cisplatin was added to the wells for 48 h. Following the indicated incubation period for the above assays, the number of viable cells was determined by measuring the A_490 _of reduced MTS solution (Promega, Madison, WI, USA). Data (triplicate experiments) are expressed as the ratio of average absorbance for treated wells to control wells, after subtracting media absorbance.

### TUNEL assay

A terminal deoxynucleotidyltransferase-mediated dUTP nick-end-labeling (TUNEL) assay was performed to measure apoptosis. Cells were cultured on 10-cm diameter dishes, and allowed to reach 50% confluence. After 24 h serum starvation, cells were treated with LY-294002 (10 μM) or DMSO for 60 min prior to rhEpo treatment (10 U/ml). After 24 h, cells were exposed to 0.5 μM cisplatin for 72 h (UMSCC-10B) or 1 μM cisplatin for 96 h (UMSCC-22B). Cells were harvested by trypsinization, fixed with 1% paraformaldehyde, and cytoplasmic DNA fragments with 3'-hydroxyl ends were detected with an APO-Direct TUNEL kit (Phoenix Flow Systems, San Diego, CA, USA).

### Statistics

Experiments were performed in triplicate and results represent mean and SD where appropriate. Statistical significance of the effect of rhEpo on proliferation, invasion, and survival was tested using a two sample independent *t*-test (2-tailed test) with the threshold set at *P *< 0.05.

## Results

### HNSCC cell lines UMSCC-10B and UMSCC-22B express EpoR and endogenous Epo

**Figure 1:**
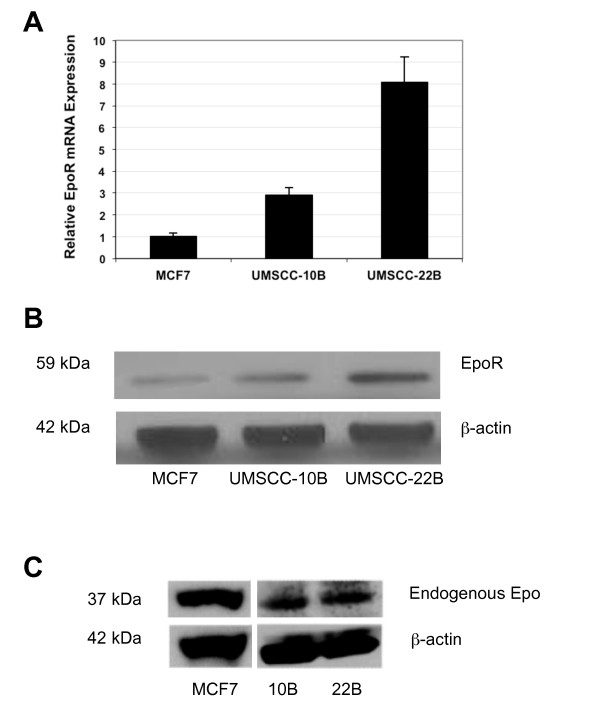
**EpoR and endogenous Epo expression in two established HNSCC cell lines**. **a **Quantitative polymerase chain reaction (qPCR) and **b **Western blot analysis demonstrating the expression of EpoR in two established cell lines. **c **Western blot analysis also indicates UMSCC-10B and UMSCC-22B express moderate levels of endogenous Epo. MCF-7 cells were used as a positive control for EpoR and endogenous Epo expression.

Both cell lines showed expression of EpoR. MCF-7 cells, which moderately express EpoR [12,13], were used as a positive control for EpoR mRNA and protein expression levels. Detected levels of EpoR mRNA in UMSCC-10B and UMSCC-22B were 2.9- and 8.1-fold higher than MCF-7, respectively. In both HNSCC cell lines, EpoR protein was expressed at relatively high levels, which correlated with mRNA data (Figure 1). In addition, moderate levels of endogenous Epo expression were detected in both HNSCC cell lines. The internal control for western blots and RT-qPCR analysis was β-Actin.

### RhEpo induces HNSCC cell proliferation

**Figure 2:**
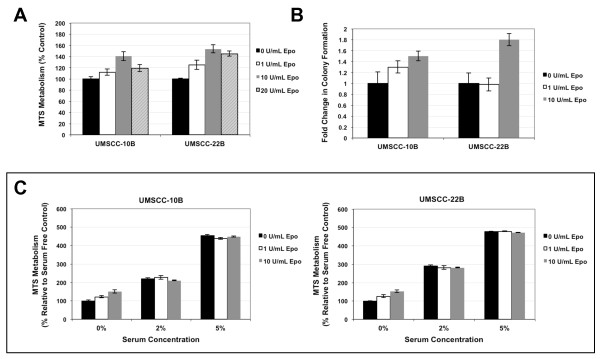
**Effects of rhEpo on cell proliferation**. RhEpo promotes moderate cell proliferation under serum free conditions as measured by **a **colorimetric MTS assay and **b **colony formation assay in UMSCC-10B and UMSCC-22B. **c **In the presence of low or moderate levels of serum, rhEpo failed to modulate cell proliferation.

Pharmacological doses of rhEpo exhibited moderate effects on cell proliferation with a maximal response at 10 U/ml. Epo at 1 U/ml increased cell proliferation by 12% and 25% in UMSCC-10B and UMSCC-22B, respectively, while 10 U/ml increased proliferation by 41% and 53% (Figure 2a). Proliferative effects of rhEpo were only apparent under serum-free conditions, and significantly less than serum stimulation (Figure 2c). Exposure of the UMSCC-10B and UMSCC-22B cell lines to rhEpo at 1 and 10 U/ml resulted in increased cell proliferation, as determined by the number of colonies that had greater than 50 cells after 7 days of culture (Figure 2b). Under normoxic conditions in the UMSCC-10B cell line, rhEpo at 1 U/ml produced a 1.3-fold increase in colony formation (mean ± SD, 161 ± 22 versus 127 ± 9.7 colonies), while rhEpo at 10 U/ml produced a 1.5-fold increase in colony formation (mean ± SD, 190.0 ± 10.7 versus 127 ± 9.7). Under similar conditions in the UMSCC-22B cell line, rhEpo at 1 U/ml showed no appreciable effects, while rhEpo at 10 U/ml resulted in a 1.8-fold induction in colony formation (54 ± 6.8 versus 30 ± 5.5). These results indicate that rhEpo increases cell proliferation in a concentration-dependent manner in UMSCC-10B and UMSCC-22B cell lines after 6-7 days of culture.

### RhEpo promotes in vitro invasion in HNSCC cell lines

**Figure 3:**
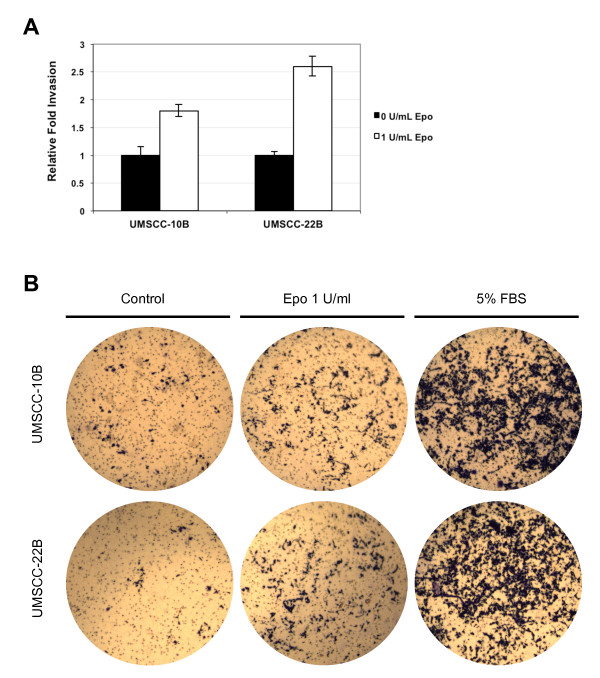
**Effects of rhEpo on invasion**. RhEpo functions as a chemoattractant for HNSCC cell lines under study, promoting in vitro invasion. **a **Exposure of cells to rhEpo at 1 U/ml for 40 h increased invasion by 80% in UMSCC10B and 160% in UMSCC-22B compared to their respective controls. **b **Images of representative fields showing an increase in the number of Matrigel invading cells upon administration of rhEpo, although less significant than serum stimulation. Magnification = 100 ×.

For invasion assay, all treatments were performed with three inserts. Addition of rhEpo at 1 U/ml increased cell invasion by 1.8-fold in the UMSCC10B cell line and 2.6-fold in the UMSCC-22B cell line compared with control (Figure 3). The effect of rhEpo on cell invasion was significant (*P *< 0.05) at a concentration of 1 U/ml, although substantially less than serum stimulation (Figure 3b). These findings indicate that exposure of the established HNSCC cell lines to rhEpo for 40 h can increase cell invasion capabilities, consistent with findings reported by other investigators that used the UMSCC-22B cell line.

### RhEpo protects HNSCC cells from cisplatin-induced cell death

**Figure 4:**
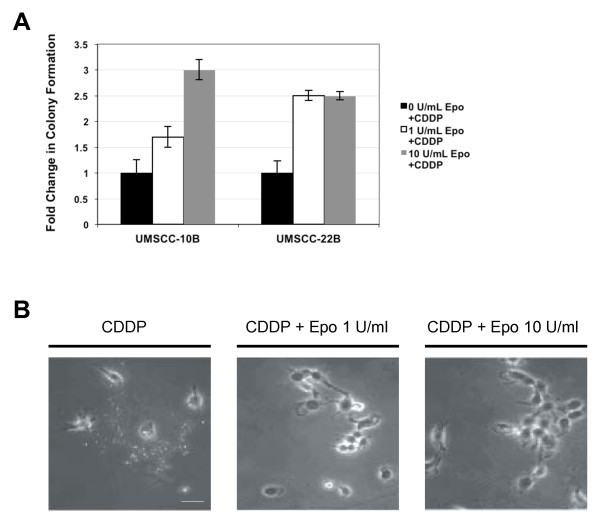
**RhEpo protects HNSCC cell lines against cisplatin-induced cell death**. **a **Clonogenic assay demonstrating that in UMSCC-10B cells treated with 0.5 μM cisplatin, exposure to rhEpo at 1 and 10 U/ml resulted in a 1.7 ± 0.2 fold and 3.0 ± 0.2 fold increase in colony number, compared to control cells not exposed to rhEpo, respectively. In cell line UMSCC-22B treated with 1.0 μM cisplatin, rhEpo at 1 U/ml resulted in a 2.5 ± 0.1 fold increase in colony number compared to the control cells. **b **Micrographs of representative fields showing the cytoprotective effects of rhEpo on cell morphology after cisplatin treatment. Shrunken cytosol and fragmentation of cells into apoptotic bodies, depicted in the far left micrograph, was noticeably reduced upon exposure to rhEpo at 1 and 10 U/ml. Scale bar represents 50 μm.

In the UMSCC-10B cells treated with 0.5 μΜ cisplatin, exposure to rhEpo at 1 and 10 U/ml resulted in a 1.7 ± 0.2 fold (16.25 ± 3.3 colonies) and 3.0 ± 0.2 fold (29.0 ± 5.3 colonies) increase (*P *= 0.06) in colony number, respectively, compared to control cells not exposed to rhEpo (9.75 ± 2.4 colonies). In the UMSCC-22B cell line treated with 1.0 μM cisplatin, rhEpo at 1 U/ml resulted in a 2.5 ± 0.1 fold (26.8 ± 1.6 colonies versus 10.5 ± 1.9 colonies) increase in colony number (*P *< 0.05) compared to the control cells, while rhEpo at 10 U/ml resulted in a 2.4 ± 0.1 fold (24.6 ± 1.8 colonies versus 10.5 ± 1.9 colonies) increase in colony number (*P *< 0.05) compared to the control cells (Figure 4). These results indicate that rhEpo protects HNSCC cells against cisplatin.

### Inhibition of PI3K/Akt pathway mitigates rhEpo-mediated cytoprotective effects

**Figure 5:**
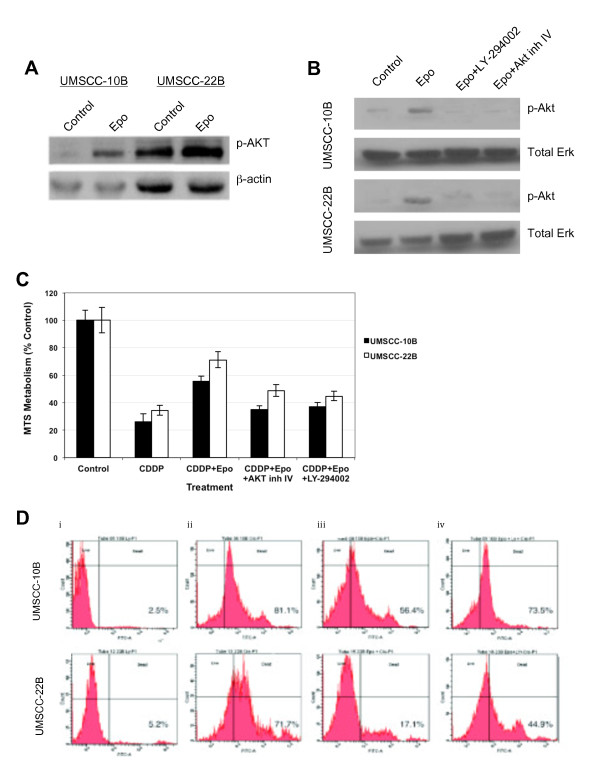
**Akt activation by PI3K is involved in rhEpo-mediated cisplatin resistance**. **a **Western blot analysis demonstrating that exposure to 1 U/ml of rhEpo induces Akt activation in both UMSCC-10B and UMSCC-22B cell lines which is sustained for at least 72 h. **b **RhEpo-mediated Akt phosphorylation is dependent on PI3K, as determined by western blot analysis. Cells were pre-treated with either drug vehicle, 10 μM LY-294002, or 1.25 μM Akt inhibitor IV for 60 min prior to rhEpo treatment for 3 h. Total Erk was used as loading control. **c **MTS assays used to measure cell viability in UMSCC-10B and UMSCC-22B upon exposure to cisplatin and rhEpo show a 39.9% and 56.0% reduction in loss of cell viability when compared to cisplatin alone, respectively. Pre-treatment with Akt inhibitor IV resulted in a 69.6% and 61.2% reduced protection of rhEpo-treated UMSCC-10B and UMSCC-22B, respectively, and treatment with LY-294002 resulted in a 63.0% and 71.7% reduced protection. **d **TUNEL assays used to measure cell death in UMSCC-10B (top panel) and UMSCC-22B (bottom panel) reveal a significant reduction in cell death when treated with rhEpo and cisplatin (iii) when compared to cisplatin alone (ii), a 30.5% and 76.2% reduction in cell death, respectively. UMSCC-10B and UMSCC-22B cells exposed to rhEpo, cisplatin, and LY-294002 (PI3K/Akt signaling inhibitor) experienced a 69.2% and 50.9% reduced protection, respectively. Cells treated with only LY-294002 (i) experienced very little cell death (2-5%) while control cells exposed to drug vehicle, cells exposed to rhEpo alone, and cells exposed to rhEpo and LY-294002, experienced less than 1% cell death.

As shown in Figure 5a and 5b, exposure to rhEpo resulted in a significant increase in Akt activation in both cell lines, which was dependent on PI3K. RhEpo-induced Akt activation was noticeable after 3 h and sustained for at least 72 h. To further investigate the role of Akt in the protective effects of rhEpo, the cell lines were exposed to cisplatin with or without rhEpo and Akt inhibitor IV, and cell viability was measured by MTS assay (Figure 5c). RhEpo protected cells from cisplatin-induced death, reducing loss of cell viability by 39.9% and 56.0% in UMSCC-10B and UMSCC-22B, respectively, compared to cisplatin alone. Pre-treatment with Akt specific inhibitor IV resulted in a 69.6% and 61.2% reduced protection of rhEpo-treated UMSCC-10B and UMSCC-22B cells exposed to cisplatin, respectively. Treatment with LY-294002 (PI3K/Akt signaling inhibitor) resulted in a similar inhibition of rhEpo-mediated cytoprotection (63.0% and 71.7% reduced protection in UMSCC-10B and UMSCC-22B, respectively). Treatment of cells with drug vehicle, Akt inhibitor IV, or LY-294002 resulted in less than 5% decrease in cell viability compared to untreated cells. In a similar experiment, a TUNEL assay was performed to measure cell death. When cisplatin was combined with rhEpo, a 76.5% reduction in cell death was observed in UMSCC-22B cells and a 30.5% reduction in cell death was observed in UMSCC-10B (*P *< 0.05) (Figure 5d). However, when cells were exposed to rhEpo, cisplatin, and 10 μM LY-294002, UMSCC-10B experienced a 9.4% reduction in cell death compared to cisplatin alone. That is, 69.4% less effective in protecting cells from cisplatin-induced cell death than rhEpo alone. Under the same conditions, UMSCC-22B experienced a 37.3% reduction in cell death compared to the cisplatin alone, about 51% less effective in protecting cells than rhEpo alone. Control cells exposed to drug vehicle, cells exposed to rhEpo, and cells exposed to rhEpo and LY-294002 experienced less than 1% cell death in both cell lines.

## Discussion

ESAs are highly effective in treating anemia, a frequent side effect of chemotherapy. But major safety issues reported in recent clinical trials have dampened the enthusiasm in the use of ESAs, and have raised legitimate concerns regarding the routine use of ESAs for treatment of anemia in cancer patients. For instance, two trials that evaluated the potential for ESAs to improve overall or progression-free survival in cancer patients reported in 2003 an increased risk of mortality in patients with breast cancer who were treated with ESA and chemotherapy [4], as well as poor survival in patients with HNSCC who received ESA and radiotherapy [5]. Other published reviews of safety information for ESAs have also raised concerns about increased tumor progression and mortality in patients administered ESAs [6,7,14,15]. Although rhEpo has been implicated in the regulation of tumor growth, the precise role of rhEpo/EpoR in human cancers is not well understood.

In the present study, we utilized two established HNSCC cell lines to characterise the contribution of rhEpo/EpoR signaling to cell proliferation, invasion and apoptosis. Both cell lines were shown to express EpoR by qPCR and western blot analysis. EpoR protein was expressed at relatively high levels in both cell lines, which was confirmed by mRNA data. EpoR expression was higher in UMSCC-22B than UMSCC-10B cell line. The difference in EpoR expression between the two cell lines may be related to the slightly higher tumor grade of UMSCC-22B [16,17]. It should be pointed out that the selectivity/specificity of antibodies used for the detection of functional EpoR is an important consideration. It seems the specificity of commercial EpoR antibodies is under speculation. However, Elliott et al. has recently demonstrated that the M-20 antibody is capable of detecting EpoR through western blot analysis [12].

The effect of rhEpo on cell proliferation was investigated through MTS and clonogenic assays. Our findings indicate that rhEpo increases proliferation in a concentration-dependent manner in UMSCC-10B and UMSCC-22B cell lines at pharmacologic doses. As these cell lines showed high expression of EpoR and enhanced proliferative ability under rhEpo exposure, it is likely that the rhEpo effects are mediated through the activity of EpoR. Lai et al. reported a limited effect on HNSCC proliferation at the 1 U/ml dose, while higher pharmacologic doses (10-100 U/ml) of rhEpo were required to achieve a measurable proliferation response [9]. Other investigators have found only a limited or no effect on cell proliferation upon exposure to rhEpo by evaluating EpoR positive cell lines, human melanoma cells, or other non-hematopoietic cancer cell lines [18-20]. This suggests that the proliferative effects of rhEpo may be cell type specific and dependent on whether cells express functional Epo receptors. A study by Hardee et al. [21] similarly showed no proliferative advantage to a HNSCC cell line FaDu when exposed to rhEpo in vivo. The lack of response may be attributed to low or no expression of EpoR, as the EpoR levels in FaDu are unclear. Also, during the in vivo experiments, it is notable that rhEpo was administered only after a 200 mm^3 ^tumor was achieved [21]. We hypothesize that rhEpo-induced cell proliferation may be restricted to stages of initial tumor development.

The results of our invasion assay showed that exposure of the established cell lines to rhEpo induced a more robust invasion in HNSCC cells. This finding is consistent with the results reported by Lai et al. and Mohyeldin et al. who demonstrated that rhEpo promotes invasion using a Matrigel invasion assay [9,10]. The increased invasion was shown by both investigators to be through the Janus kinase-Signal transducer and transcription (JAK-STAT) pathway [9,10]. As the majority of head and neck cancer related morbidity is a result of local invasion and extension of the solid tumor, these findings indicate that rhEpo-induced invasion may have contributed to the primary or secondary outcome measures of the HNSCC patients trial [5], in which patients experienced increased locoregional recurrence and decreased survival when treated concomitantly with rhEpo [5]. In another study, EpoR expression in neuroblastoma primary tumors has been shown to have significantly lower expression when compared to paired lymph node metastases, a further indication that EpoR is highly implicated in metastasis [22].

Coexpression of EpoR and endogenous Epo has been detected in a variety of primary cancers and tumor cell lines, including non-small cell lung cancer, breast cancer, and cervical cancer [1]. In certain cancers, such as uterine, ovarian, melanoma, and stomach choriocarcinoma, inhibition of this autocrine/paracrine Epo/EpoR signaling pathway altered critical aspects of tumor biology, including inhibited proliferation and increased apoptotic cell death [1]. Our data demonstrating endogenous Epo expression in UMSCC-10B and UMSCC-22B indicates the possible existence of an Epo/EpoR autocrine/paracrine neoplastic pathway which promotes malignant progression of HNSCC, further propagated by administration of exogenous rhEpo. As a result, the limited effect on cell proliferation and invasion of exogenously added rhEpo may be a consequence of the moderately high basal levels of Epo present in both cell lines. Thus, in the absence of endogenous Epo, the pharmacological doses used in this study may have induced a more pronounced effect on cell growth and invasion than observed. Further studies should be devoted to studying the effects of endogenous Epo expression on regulating a malignant phenotype in HNSCC.

In addition to promoting cell proliferation and invasion, it is also possible that rhEpo inhibits apoptosis in cancer cells. RhEpo has been shown to induce anti-apoptotic genes including *Bcl-xL*, *Bcl-2*, and *Mcl-1 *in Ewing sarcoma and neuroblastoma cell lines [23]. It has also been reported that rhEpo decreased apoptosis when melanoma cells were exposed to darcarbazine and cispatin, and increased the surviving fraction of cervical carcinoma cells treated with cisplatin [24,25]. Belenkov et al. also reported resistance of malignant glioma and primary cervical cancer lines to radiation and cisplatin-induced cell death upon addition of rhEpo [26]. This finding was mitigated and reversed upon addition of a Jak2 inhibitor [26]. More recently, it has been demonstrated that both hypoxia and rhEpo protect glioblastoma multiform cells from cisplatin cytotoxicity [27]. In contrast, others have demonstrated that rhEpo sensitizes human renal cell carcinoma and myelomonocytic leukemia cell lines to daunorubicin and vinblastine through inhibition of the NF-kappa b pathway [28]. Furthermore, Palumbo et al. showed that rhEpo fails to modulate pemetrexed or cisplatin sensitivity of EpoR expressing mesothelioma cell lines, despite phosphorylating Akt [29].

We are the first to address the specific in vitro effects of rhEpo on HNSCC survival when administered together with cisplatin, using colony formation assays. These experiments are especially important, as the colony formation assay is most relevant in determining the long-term protective effects of rhEpo, particularly when clinical doses of rhEpo and cisplatin are used. Our study indicates that the addition of rhEpo mitigates the pro-apoptotic effects of cisplatin, rendering this first-line HNSCC drug significantly less effective. The intracellular mechanism of the Epo ligand binding to its receptor is well documented. EpoR is a ubiquitous membrane receptor, and when Epo binds, the EpoR receptor homodimerizes, regulating activation of the PI3K/Akt signal transduction pathway [30]. We further investigated the potential role of Akt in the protective effects of rhEpo. Exposure to rhEpo resulted in a significant increase in Akt activation in both cell lines. The fact that direct inhibition of Akt produced results comparable to PI3K inhibition (with LY-294002) indicates that the observed effects of LY-294002 are due to interruption of the PI3K/Akt signaling pathway. Collectively, the data implicates Akt activation in the cytoprotective effects of rhEpo against cisplatin-induced death. However, as the PI3K and Akt inhibitors did not completely block the cytoprotective effects of rhEpo, it is likely that rhEpo activation of other signaling pathways, such as JAK2/STAT5, contributes to the observed cisplatin resistance.

Our results suggest p-Akt may play a pivotal role in the protective effects of rhEpo. This is consistent with the findings of several groups that rhEpo's effects are mediated in part through the PI3K/Akt pathway [31-33]. Further investigation is needed to elucidate the role of PI3K/Akt signaling in rhEpo-induced resistance.

## Conclusions

The results demonstrate that, in HNSCC cells expressing functional EpoR, rhEpo promotes invasion, cell proliferation, and induces resistance to cisplatin, which may contribute to tumor progression. Modulation of the response of HNSCC cells to cisplatin may significantly contribute to the adverse effects seen in HNSCC patients receiving rhEpo. Given the results of this study and the broad signaling of the EpoR cascade, it is unlikely that the decrease in patient survival can be attributed to a single source. Currently, the relative importance of these mechanisms is yet to be elucidated. We propose further studies to investigate the effect of rhEpo in vivo in xenograft mouse models to determine the relative effects of these mechanisms.

## Abbreviations

rhEpo: Erythropoietin; EpoR: Erythropoietin receptor; HNSCC: Head and neck squamous cell carcinoma; PI3K: Phosphatidylinositol 3-kinase.

## Competing interests

The authors declare that they have no competing interests.

## Authors' contributions

EA carried out the TUNEL assay, western blots, figure preparation, and wrote the first draft of the manuscript. ER carried out the qPCR experiment, western blots, invasion assays, MTS assays, figure preparation, and wrote the second draft of the manuscript. JW participated in its design and coordination and helped draft the manuscript. KJB carried out the clonogenic assays and helped draft the manuscript. MY carried out the invasion assays and helped with figure preparation. RW participated in its design and coordination and helped draft the manuscript. KB participated in its design and coordination and helped draft the manuscript. WO conceived of the study, participated in its design and coordination and helped draft the manuscript.
